# Characterization of a *Neisseria gonorrhoeae* Ciprofloxacin panel for an antimicrobial resistant Isolate Bank

**DOI:** 10.1371/journal.pone.0264149

**Published:** 2022-03-10

**Authors:** Hsi Liu, Kevin Tang, Cau D. Pham, Matthew Schmerer, Ellen N. Kersh, Brian H. Raphael

**Affiliations:** 1 Division of STD Prevention, National Center for HIV/AIDS, Viral Hepatitis, STD, and TB Prevention, Centers for Disease Control and Prevention, Atlanta, Georgia, United States of America; 2 Division of Scientific Resources, National Center for Emerging and Zoonotic Infectious Diseases, Centers for Disease Control and Prevention, Atlanta, Georgia, United States of America; Cornell University, UNITED STATES

## Abstract

**Objectives:**

*Neisseria gonorrhoeae* (gonococcus) infection is one of the most commonly reported nationally notifiable conditions in the United States. Gonococcus has developed antimicrobial resistance to each previously used antibiotic for gonorrhea therapy. However, some isolates may be still susceptible to no longer recommended, yet still effective antibiotics. This in turn suggests that targeted therapy could slow resistance development to currently recommended empirical treatments. We curated a gonococcal Ciprofloxacin Antibiotic Resistance Isolate Bank panel (Cipro-panel) as a tool for validating or developing new tests to determine ciprofloxacin susceptibility.

**Method:**

The Cipro-panel was selected using whole genome sequencing, bioinformatic tools, and antimicrobial susceptibility testing (AST) data. Isolates were further selected based on nucleotide variations in *gyrA* and *parC* genes.

**Results:**

We selected 14 unique *N*. *gonorrhoeae* isolates from the 2006–2012 Gonococcal Isolate Surveillance Project (GISP) collection. They represented a wide range of antimicrobial susceptibility to ciprofloxacin and commonly observed nucleotide variations of *gyrA* and *parC* genes. This Cipro-panel consists of 5 isolates with resistant phenotypes (MIC > = 1 μg/mL), 8 isolates with susceptible phenotypes (MIC < = 0.06 μg/mL), and 1 isolate falling in the Clinical and Laboratory Standards Institute defined intermediate range. Among the *gyrA* variations we observed a total of 18 SNPs. Four positions had nonsynonymous changes (nucleotide positions 272, 284, 1093, and 1783). The first two positions (272 and 284) have been linked previously with resistance to ciprofloxacin (i.e. amino acid positions 91 and 95). For the *parC* gene, we observed a total of 21 possible SNPs. Eight of those SNPs resulted in non-synonymous amino acid changes. One location (amino acid 87) has been previously reported to be associated with ciprofloxacin resistance.

**Conclusions:**

This Cipro-Panel is useful for researchers interested in developing clinical tests related to ciprofloxacin. It could also provide additional choices for validation, quality assurance purposes and improve antibiotic usage.

## Introduction

*Neisseria gonorrhoeae* (gonococcus) is the causative agent of gonorrhea, one of the nationally notifiable conditions in the United States and one of the most commonly reported Sexually Transmitted Diseases in the world [1, 2]. *Neisseria gonorrhoeae* is an exclusive human pathogen and is well-adapted to the genital system of the human. However, it can cause infections in both male and female reproductive systems, the pharynx, the rectum, and other anatomical sites (e.g. joints) as a disseminated infection. The organism is genetically versatile in its ability to develop drug resistance, thus development of resistance to antimicrobial agents by the gonococcus is a major public health concern. In the past, while retaining previously acquired antimicrobial resistance, *N*. *gonorrhoeae* developed resistance toward all first-line drugs used in the standard treatment. These drugs include commonly used antibiotics such as penicillin, tetracycline, macrolides, and fluoroquinolones such as ciprofloxacin [3–7]. Because of the gradual increase in proportion of isolates with higher minimum inhibitory concentrations (MIC) toward the current first line class of drug, cephalosporins, in 2012 CDC recommended use of dual-antibiotic therapy consisting of an injectable cephalosporin (ceftriaxone) and one oral dose of a macrolide (azithromycin) as the regimen to treat uncomplicated gonococcal infections [2, 4]. At the end of 2020, Centers for Disease Control and Prevention’s (CDC) new treatment guidelines removed azithromycin from its recommendations partly because the percentage of *N*. *gonorrhoeae* isolated with reduced susceptibility to azithromycin (MIC ≥2.0 μg/mL) increased more than sevenfold over 5 years (from 0.6% in 2013 to 4.6% in 2018). Ceftriaxone is now recommended as a monotherapy for non-complicated gonorrhea [5].

The fluoroquinolone class antibiotic ciprofloxacin was recommended by CDC’s treatment guideline as the first-line treatment option for gonorrhea from 1996 to 2006. Despite the fact that some states in the United States such as Hawaii and California have discontinued the use of ciprofloxacin for gonorrhea treatment, however, not until 2007 ciprofloxacin was discontinued recommended by CDC as the first option for treating non-complicated gonorrhea [3, 4]. The principle for this decision was based on a recommendation by the WHO that when the microbial resistance rate toward an antibiotic reaches 5% in the population, the drug may be removed from use [8–11]. Fluoroquinolones exert their activity by inhibiting the replication of gonococci through interference of the binding of DNA gyrase and topoisomerase. Drug resistance toward ciprofloxacin thus developed through mutations in DNA gyrase (GyrA) and topoisomerase IV subunits (ParC) [6, 7, 12].

In response to the concerns of increasing antibiotic resistant isolates, CDC published a threat report ranking drug resistant *N*. *gonorrhoeae* as “Urgent Threat” [9]. An important effort is to develop tools that can enhance the detection of antibiotic resistance in gonorrhea locally, nationally, and internationally. To this end, a *N*. *gonorrhoeae* Ciprofloxacin Antibiotic Resistance Isolate Bank panel (Cipro-panel) was curated and made available through the CDC & FDA AR Isolate Bank (AR Bank) [13]. In this panel, isolates were whole genome sequenced, characterized, and susceptibility to ciprofloxacin was documented. We hope this panel can help developing advanced point of care tests to quickly identify infections that are still susceptible to ciprofloxacin.

## Methods

### Bacteria strains

*N*. *gonorrhoeae* isolates were propagated on GC base medium with 1% IsoVitalex and 5% FBS (SRP, Scientific Resources Program, CDC) at 36±1°C supplemented with 5% CO_2_ for 20 to 24 hours. A 300–500 ul culture in trypticase soy broth (TSB) containing 20% glycerol (SRP, CDC) was kept at -70°C. Isolates included in this Cipro-panel were characterized using standard microbiological methods. Species identification was confirmed using the AP-NHI strips (Analytical Profile Index for *Neisseria* and *Haemophilus*; bioMerieux, France). The species identification of each isolate was further verified using matrix-assisted laser desorption-ionization time of flight mass spectrometry (MALDI-TOF) following manufacturer’s recommendation (Bruker Microflex Biotyper, Billerica, MA) [14].

### Antimicrobial susceptibility testing (AST)

The ciprofloxacin agar dilution method was performed according to the Clinical and Laboratory Standards Institute (CLSI) M07 protocol [15, 16] and following the Clinical Laboratory Improvement Amendments (CLIA) regulations. The Etest method was used as additional verification and performed as previously described [17]. The breakpoints and determination of susceptibility (S), intermediate range (I), and resistance (R) were based on CLSI criteria M100 [16]. In brief, the agar dilution and Etest methods were prepared by suspending colonies of *N*. *gonorrhoeae* from an overnight Chocolate II agar plate (SRP, CDC) into Mueller-Hinton broth (Difco Laboratories, Fisher Scientific, MI) and adjusted to an optical density equal to that of a 0.5 McFarland standard. The cultures were applied to plates of specific antibiotic concentrations (agar dilution) or streaked to a plate and appropriate antibiotic strips (bioMerieux, France) were applied (Etest). The plates were incubated at 36±1°C in 5% CO2 for 20–24 hours. The minimum inhibitory concentrations (MICs) were interpreted by reading growth inhibition (agar dilution) or the intercept of the inhibition zone around the strip (Etest). The agar dilution MIC values were reported and Etest was used for verification purpose.

### Whole-genome sequencing and analyses

DNA was extracted using the Promega Genomic DNA Purification Kit (Promega, Madison, WI) and whole-genome sequencing was performed using a standard protocol [18, 19]. Specifically, libraries were prepared using the NEB Genome Library Preparation Kit (New England Biolab, MA) and sequenced as paired-end 2x250 bp reads using the Illumina MiSeq platform (Illumina, CA). Preprocessing assessed the read quality with Trim_Galore (v 0.3.7) which contains FastQC and Cutadapt [20] to perform quality assessment, remove duplicate reads and trimming of reads. The quality of the genome was evaluated using QUAST (v 4.3) and assembled using SPAdes (v 3.9.0) [21, 22]. Finally, annotation was completed using NCBI’s Prokaryotic Genome Annotation Pipeline [23, 24]. Reads were mapped to the FA1090 reference sequence (GenBank accession number NC002946). SAMtools was used to convert the alignments and using GATK IndelRealigner command. Pilon was further used to call the variants and raw variants were filtered by using snpSift with depth > = 20 and genotype quality score > = 200. Additionally, *gyrA* and *parC* sequences were aligned and compared using the CLC Genomics Workbench software (Version 11.3, Qiagen) and Geneious Prime (V12, Qiagen). The assembly metrics and quality control data for WGS results are shown in Table 1.

**Table 1:** Genome sequencing metrics for isolates in the Cipro panel.

Cipro Panel ID	AR Bank ID	NCBI SRA Accession (raw reads)	Genome Assembly Accession Number	Total_ reads	Read_ length (bp)	No_ contigs	GC_ content (%)	N50 (bp)	Assembled_ genome_ length (bp)	Isolate_coverage (x)
1	963	SRR8833284	SUQT00000000	1231842	251	85	52.51	67319	2161799	140.541974
2	964	SRR8833283	SUQW00000000	1659350	251	74	52.42	68575	2167268	189.31675
3	965	SRR8833297	SUQX00000000	1865032	251	120	52.54	75579	2187389	212.783196
4	966	SRR13058147	SUQY00000000	1243062	251	81	52.62	65729	2103800	141.822074
5	967	SRR8833292	SUQZ00000000	1256404	251	102	52.53	72659	2169573	143.344275
6	968	SRR8833294	SURA00000000	1422260	251	75	52.32	84733	2220872	162.266936
7	969	SRR8833282	SURC00000000	1535756	251	133	52.51	72330	2239672	175.215798
8	970	SRR8833288	SURE00000000	1432304	251	74	52.64	57643	2104215	163.412865
9	971	SRR8833289	SURG00000000	981884	251	83	52.34	68047	2199798	112.024038
10	972	SRR13058146	SURH00000000	977638	251	74	52.4	76548	2171544	111.539608
11	973	SRR8833290	SURI00000000	1593064	251	89	52.32	59588	2197787	181.75412
12	974	SRR8833291	SURJ00000000	1265524	251	71	52.41	71271	2157111	144.384784
13	975	SRR8992476	VAHK00000000	986758	251	87	52.4	57773	2165232	112.580117
14	976	SRR8992477	VAHL00000000	1319478	251	74	52.43	86811	2171852	150.540445

## Results

### Selection criteria

Two hundred fifty newly sequenced *N*. *gonorrhoeae* isolates from the United States’ Gonococcal Isolate Surveillance Project (GISP; [25, 26]) collected from year 1999 to 2012 and archived at the CDC were used to select this panel. Based on the unique *gyrA* and *parC* sequence variations together with their ciprofloxacin susceptibility profile, 14 unique isolates were selected and included in this Ciprofloxacin Antibiotic Resistance Isolate Bank panel (Cipro-panel). The isolates included in this Cipro- panel are numbered from 1 to 14 with corresponding AR Bank numbers 963–976. Their MIC values and the accession numbers of the whole genome sequencing results are available online (Table 1) [13].

### Genotype characterizations

We observed two well-described mutations in *gyrA* at positions 272 and 284, which caused non-synonymous changes corresponding to amino acid 91 and 95, respectively. We also observed additional mutations at 12 nucleotide positions (Table 2). They are at nucleotide positions, 276, 279, 666, 744, 882, 927, 1032, 1094, 1110, 1722, 1783, and 2433. Among these, positions 1094 and 1783 resulted in amino acid changes. They are arginine to histidine at position 365 (R365H) of panel number 8, and alanine to threonine at position 595 (A595T) of panel number 5. There were no known functional changes associated with these additional amino acid mutations.

**Table 2:** Observed *gyrA* mutations.

Bank No			Amino Acid mutation				Location of Nucleotide Mutation												
Cipro Panel ID	AR Bank ID	CIP MIC (μg/ml)	gyrA aa91	gyrA aa95	parC aa87	TCC 272	GCA 276	GTT 279	GAC 284	GCC 666	GTT 744	ACA 882	GGT 927	GTG 1032	CGC 1094	GTC 1110	GGC 1722	GCC 1783	CCG 2433
1	963	0.5	F	G	S	TTC	GCA	GTT	GGC	GCC	GTT	ACA	GGT	GTT	CGC	GTC	GGC	GCC	CCG
2	964	32	F	G	R	TTC	GCA	GTT	GGC	GCC	GTC	ACA	GGT	GTG	CGC	GTG	GGC	GCC	CCG
3	965	0.004	S	D	S	TCC	GCA	GTT	GAC	GCC	GTC	ACG	GGG	GTT	CGC	GTC	GGC	GCC	CCG
4	966	0.008	S	D	S	TCC	GCA	GTT	GAC	GCC	GTC	ACG	GGG	GTT	CGC	GTC	GGC	GCC	CCA
5	967	0.008	S	D	S	TCC	GCA	GTT	GAC	GCC	GTC	ACG	GGG	GTT	CGC	GTC	GGC	ACC	CCG
6	968	0.004	S	D	S	TCC	GCA	GTT	GAC	GCA	GTT	ACA	GGT	GTG	CGC	GTC	GGC	GCC	CCG
7	969	0.004	S	D	S	TCC	GCC	GTA	GAC	GCC	GTT	ACG	GGG	GTT	CGC	GTC	GGC	GCC	CCG
8	970	0.004	S	D	S	TCC	GCA	GTT	GAC	GCC	GTT	ACG	GGG	GTT	CAC	GTC	GGC	GCC	CCG
9	971	8	F	A	S	TTC	GCA	GTT	GCC	GCC	GTT	ACA	GGT	GTG	CGC	GTC	GGC	GCC	CCG
10	972	0.008	S	D	S	TCC	GCA	GTT	GAC	GCC	GTT	ACA	GGT	GTG	CGC	GTC	GGT	GCC	CCG
11	973	0.004	S	D	S	TCC	GCA	GTT	GAC	GCC	GTT	ACA	GGT	GTG	CGC	GTC	GGC	GCC	CCG
12	974	32	F	G	R	TTC	GCA	GTT	GGC	GCC	GTC	ACG	GGG	GTT	CGC	GTC	GGC	GCC	CCG
13	975	32	F	G	R	TTC	GCA	GTT	GGC	GCC	GTC	ACA	GGT	GTG	CGC	GTC	GGC	GCC	CCG
14	976	32	F	G	R	TTC	GCA	GTT	GGC	GCC	GTC	ACA	GGT	GTG	CGC	GTC	GGC	GCC	CCG

The *parC* gene has mutations in 19 nucleotide locations (Table 3) which include the commonly recognized AGT to CGT at position 259 (S87R). Several non-synonymous amino acid changes resulting from nucleotide changes other than position 259 were also recognized. They are at positions 1150 (I384V), 1304/5 (V436A), 1375 (G459S), 1435 (L479F), 1789(V596I), and 1912 (M637I). There were no known functional changes associated with these additional amino acid mutations.

**Table 3:** Observed *parC* mutations.

Bank No			Amino Acid mutation				Location of Nucleotide Mutation																	
Cip Panel ID	AR Bank ID	CIP MIC (μg/ml)	gyrA aa91	gyrA aa95	parC aa87	AGT 259	TAC 312	GCG 387	CTC 393	CTG 414	AAT 819	GGC 876	AAG 879	CTT 903	CCG 990	ATC 1150	GTG 1304/05	CAG 1326	TTG 1360	GGT 1375	AAT 1431	CTT 1435	GTT 1789	ATG 1912
1	963	0.5	F	G	S	AGT	TAT	GCG	CTC	CTG	AAT	GGC	AAG	CTT	CCG	ATC	GTG	CAG	TTG	GGT	AAT	TTT	GTT	ATG
2	964	32	F	G	R	CGT	TAC	GCG	CTG	CTG	AAT	GGC	AAG	CTT	CCC	GTC	GCA	CAG	CTG	GGT	AAC	CTT	GTT	ATG
3	965	0.004	S	D	S	AGT	TAC	GCG	CTG	CTG	AAT	GGT	AAA	CTC	CCC	GTC	GTG	CAA	CTG	GGT	AAC	CTT	GTT	ATG
4	966	0.008	S	D	S	AGT	TAC	GCG	CTG	CTG	AAT	GGT	AAA	CTC	CCC	GTC	GTG	CAA	CTG	GGT	AAC	CTT	GTT	ATG
5	967	0.008	S	D	S	AGT	TAT	GCG	CTG	CTA	AAC	GGC	AAG	CTT	CCG	GTC	GTG	CAA	CTG	GGT	AAT	TTT	ATT	ATG
6	968	0.004	S	D	S	AGT	TAT	GCG	CTG	CTG	AAT	GGT	AAA	CTC	CCG	GTC	GTG	CAG	TTG	AGT	AAT	TTT	ATT	ATG
7	969	0.004	S	D	S	AGT	TAT	GCG	CTC	CTG	AAC	GGC	AAG	CTT	CCG	ATC	GCA	CAG	CTG	GGT	AAT	TTT	ATT	ATG
8	970	0.004	S	D	S	AGT	TAT	GCG	CTG	CTA	AAT	GGC	AAG	CTT	CCG	ATC	GCA	CAG	CTG	GGT	AAC	CTT	GTT	ATA
9	971	8	F	A	S	AGT	TAT	GCA	CTG	CTA	AAT	GGT	AAA	CTC	CCG	GTC	GTG	CAA	CTG	GGT	AAAT	TTT	I/ATT	ATG
10	972	0.008	S	D	S	AGT	TAT	GCG	CTC	CTG	AAT	GGC	AAG	CTT	CCG	/ATC	GTG	CAG	TTG	GGT	AAT	TTT	GTT	ATG
11	973	0.004	S	D	S	AGT	TAT	GCG	CTG	CTA	AAT	GGC	AAG	CTT	CCG	ATC	GCA	CAG	CTG	GGT	AAC	CTT	GTT	ATG
12	974	32	F	G	R	CGT	TAC	GCG	CTG	CTG	AAT	GGT	AAA	CTC	CCC	GTC	GTG	CAA	CTG	GGT	AAC	CTT	GTT	ATG
13	975	32	F	G	R	CGT	TAC	GCG	CTG	CTG	AAT	GGC	AAG	CTT	CCC	GTC	GCA	CAG	CTG	GGT	AAC	CTT	GTT	ATG
14	976	32	F	G	R	CGT	TAC	GCA	CTG	CTG	AAT	GGC	AAG	CTT	CCC	GTC	GCA	CAG	CTG	GGT	AAC	CTT	GTT	ATG

### Phenotypic characterizations and antibiotic susceptibility

Traditionally the GyrA amino acid positions S91 and D95 are considered wild type and representing the ciprofloxacin susceptible phenotypes. Thus, based on CLSI AST ciprofloxacin testing criteria (M100; 17), 8 isolates have the susceptible phenotype (MIC< = 0.06 μg/mL; wild-type S91/D95: panel numbers 3, 4, 5, 6, 7, 8, 10, 11); 5 isolates have a resistant phenotype (MIC > = 1 μg/mL; panel numbers 2, 12, 13, 14, F91/G95; No 9, F91/A95), and one isolate has the intermediate phenotype (0.12 μg/mL < = MIC < = 0.5 μg/mL; panel number 1, F91/G95).

For the ParC amino acid composition, ten isolates have the ParC wild-type (S87, Table 3) and four have the mutant phenotype of R87 (No. 2, 12, 13, 14). Combined, eight isolates are wildtype for both GyrA and ParC (Nos 3, 4, 5, 6, 7, 8, 10, 11). Six isolates have a combined amino acid variation at either GyrA or ParC. Among these, number 1 has a F91/G95/S87 (here referred to as FGS) combination; numbers 2, 12, 13, and 14 have an FGR combination, and number 9 has the FAS combination.

### Supplemental genetic profiles of the Cipro-panel

A more detailed genetic mutational analyses is included in Table 4a–4f) as a supplement to this collection. This table was created using CDC’s Drug-Resistant Gonorrhea Genome Profiler version 2.9.2 [CDC, accessible online: https://amdportal-sams.cdc.gov/].

**Table 4:** Complete genetic profile based on Gonorrhea Genome Profiler v2.9.2.

**A**																
**Cip Panel ID**	**AR Bank ID**	**CIP MIC**	**PEN MIC**	**TET MIC**	**CRO MIC**	**CFM MIC**	**AZN MIC**	**GEN MIC**	**Beta-lactamase**	**23S-2611 base**	**23S-2059 base**	**23S-2059 freq**	**23S-2058 base**	**23S-2058 freq**	**mtrR mosaic**	**mtrR promoter**
1	963	0.38	16	1	0.004	0.008	0.25	4	Positive	C	A	1	A	1	FALSE	DEL
2	964	>32	2	2	0.015	0.03	0.25	4	Negative	C	A	1	A	1	FALSE	DEL
3	965	0.04	1	1	0.004	0.015	0.25	4	Negative	T	A	1	A	1	FALSE	A
4	966	0.08	0.25	1	0.004	0.008	≥16	4	Negative	C	G	1	A	1	FALSE	A
5	967	0.004	0.06	0.06	≤0.001	0.004	0.125	4	Negative	C	A	1	A	1	FALSE	A
6	968	0.004	0.25	0.5	0.002	0.008	0.125	4	Negative	C	A	1	A	1	FALSE	A
7	969	0.004	0.25	0.25	0.004	0.008	0.125	4	Negative	C	A	1	A	1	FALSE	A
8	970	0.004	0.5	0.5	0.008	0.03	0.06	4	Negative	C	A	1	A	1	FALSE	A
9	971	6	1	4	0.015	0.03	0.25	2	Negative	C	A	1	A	1	FALSE	DEL
10	972	0.004	0.25	1	0.004	0.015	2	4	Negative	C	A	1	A	1	FALSE	C
11	973	0.002	0.5	16	0.008	0.03	0.06	4	Negative	C	A	1	A	1	FALSE	A
12	974	>32	2	2	0.03	0.125	2	4	Negative	C	A	1	A	1	FALSE	DEL
13	975	16	2	2	0.015	0.03	0.25	4	Negative	C	A	1	A	1	FALSE	DEL
14	976	32	2	2	0.015	0.03	0.25	4	Negative	C	A	1	A	1	FALSE	DEL

**B**																
**Cip Panel ID**	**AR Bank ID**	**mtr120 promoter**	**mtrR -35**	**mtrR aa39**	**mtrR aa44**	**mtrR aa45**	**mtrR aa47**	**mtrR aa79**	**mtrR aa105**	**mtrR premature stop**	**penA allele**	**penA aa311**	**penA aa312**	**penA aa316**	**penA aa483**	**penA aa501**
1	963	G	G	A	R	D	L	D	H	FALSE	2.001	A	I	V	T	A
2	964	G	G	A	R	G	L	D	Y	FALSE	12.001	A	I	V	T	A
3	965	G	G	A	R	D	L	D	H	FALSE	2.001	A	I	V	T	A
4	966	G	G	A	R	D	L	D	H	FALSE	2.001	A	I	V	T	A
5	967	G	G	A	R	G	L	N	Y	FALSE	15.001	A	I	V	T	A
6	968	G	G	A	R	G	L	D	H	TRUE	22.001	A	I	V	T	A
7	969	G	G	A	R	G	L	N	Y	FALSE	2.002	A	I	V	T	A
8	970	G	G	A	R	D	L	D	H	FALSE	9.001	A	I	V	T	A
9	971	G	G	A	R	G	L	D	Y	FALSE	43.001	A	I	V	T	V
10	972	G	G	A	R	G	L	N	H	FALSE	2.001	A	I	V	T	A
11	973	G	G	A	R	G	L	D	H	FALSE	19.001	A	I	V	T	A
12	974	G	G	A	R	G	L	D	Y	FALSE	42.001	A	M	T	T	P
13	975	G	G	A	R	G	L	D	Y	FALSE	12.001	A	I	V	T	A
14	976	G	G	A	R	G	L	D	Y	FALSE	12.001	A	I	V	T	A

**C**														
**Cip Panel ID**	**AR Bank ID**	**penA aa512**	**penA aa542**	**penA aa545**	**penA aa549**	**penA aa551**	**penA D345ins**	**ponA aa375**	**ponA aa421**	**pilQ full length**	**pilQ aa341**	**pilQ aa526**	**pilQ aa648**	**pilQ aa666**
1	963	N	G	G	A	P	TRUE	A	P	TRUE	N	D	N	E
2	964	N	G	G	A	S	TRUE	A	P	TRUE	N	D	N	E
3	965	N	G	G	A	P	TRUE	A	L	TRUE	N	D	N	E
4	966	N	G	G	A	P	TRUE	A	L	TRUE	N	D	N	E
5	967	N	G	G	A	P	FALSE	A	L	TRUE	N	G	S	E
6	968	N	G	G	A	P	TRUE	A	L	TRUE	S	D	N	E
7	969	N	G	G	A	P	TRUE	A	L	TRUE	S	D	S	E
8	970	N	G	G	A	L	TRUE	A	L	TRUE	N	D	N	E
9	971	N	G	G	A	P	TRUE	A	P	TRUE	S	D	S	E
10	972	N	G	G	A	P	TRUE	A	L	TRUE	N	D	N	E
11	973	N	G	G	A	P	TRUE	A	P	FALSE	S	D	N	E
12	974	Y	G	S	A	P	FALSE	A	P	TRUE	N	D	N	E
13	975	N	G	G	A	S	TRUE	A	P	TRUE	N	D	N	E
14	976	N	G	G	A	S	TRUE	A	P	TRUE	N	D	N	E

**D**															
**Cip Panel ID**	**AR Bank ID**	**gyrA aa91**	**gyrA aa92**	**gyrA aa95**	**parC aa86**	**parC aa87**	**parC aa88**	**parC aa91**	**blaTEM present**	**TeTM present**	**porB allele**	**rpsJ aa57**	**ftsX aa31**	**rplD aa68**	**rplD aa70**
1	963	F	A	G	D	S	S	E	TRUE	FALSE	8	M	T	G	G
2	964	F	A	G	D	R	S	E	FALSE	FALSE	8	M	T	G	G
3	965	S	A	D	D	S	S	E	FALSE	FALSE	3	M	T	G	G
4	966	S	A	D	D	S	S	E	FALSE	FALSE	3	M	T	G	G
5	967	S	A	D	D	S	S	E	FALSE	FALSE	3	V	T	G	G
6	968	S	A	D	D	S	S	E	FALSE	FALSE	3	M	T	G	G
7	969	S	A	D	D	S	S	E	FALSE	TRUE	1	V	T	G	G
8	970	S	A	D	D	S	S	E	FALSE	FALSE	100	M	T	G	G
9	971	F	A	A	D	S	S	E	FALSE	TRUE	4	M	T	G	G
10	972	S	A	D	D	S	S	E	FALSE	FALSE	1	M	T	G	G
11	973	S	A	D	D	S	S	E	FALSE	TRUE	14	M	T	G	G
12	974	F	A	G	D	R	S	E	FALSE	FALSE	11	M	T	G	G
13	975	F	A	G	D	R	S	E	FALSE	FALSE	8	M	T	G	G
14	976	F	A	G	D	R	S	E	FALSE	FALSE	23	M	T	G	G

**E**																
**Cip Panel ID**	**AR Bank ID**	**rplV ins**	**macA aa99**	**mtrD aa42**	**mtrD aa46**	**mtrD aa48**	**mtrD aa101**	**mtrD aa174**	**mtrD aa612**	**mtrD aa662**	**mtrD aa714**	**mtrD aa821**	**mtrD aa823**	**mtrD aa826**	**macA promoter**	**norM promoter**
1	963	FALSE	D	T	H	I	N	R	F	V	R	S	K	A	C	G
2	964	FALSE	D	T	H	I	N	R	F	V	R	S	K	A	C	G
3	965	FALSE	D	T	H	I	N	R	F	V	R	S	K	A	C	G
4	966	FALSE	D	N	R	T	D	R	F	I	R	A	E	G	C	G
5	967	FALSE	N	T	H	I	N	R	F	V	R	S	K	A	C	G
6	968	FALSE	N	T	H	I	N	R	F	V	R	S	K	A	C	G
7	969	FALSE	D	T	H	I	N	R	F	V	R	S	K	A	C	G
8	970	FALSE	D	T	H	I	N	R	F	V	R	S	K	A	C	G
9	971	FALSE	D	T	H	I	N	R	F	V	R	S	K	A	C	G
10	972	FALSE	D	N	R	T	D	R	F	I	R	A	E	G	C	G
11	973	FALSE	D	T	H	I	N	R	F	V	R	S	K	A	C	G
12	974	FALSE	D	T	H	I	N	R	F	V	R	S	K	A	C	G
13	975	FALSE	D	T	H	I	N	R	F	V	R	S	K	A	C	G
14	976	FALSE	D	T	H	I	N	R	F	V	R	S	K	A	C	G

**F**																
**Cip Panel ID**	**AR Bank ID**	**ermB present**	**ermC present**	**ermF present**	**mefA present**	**gyrB aa429**	**gyrB aa450**	**acnB aa348**	**acnB aa371**	**16S-1053 base**	**16S-1053 freq**	**16S-1186 base**	**16S-1186 freq**	**rpsE aa24**	**MLST**	**NG-MAST**
1	963	FALSE	FALSE	FALSE	FALSE	D	K	G	Q	G	1	C	1	T	7367	6842
2	964	FALSE	FALSE	FALSE	FALSE	D	K	G	Q	G	1	C	1	T	1901	225
3	965	FALSE	FALSE	FALSE	FALSE	D	K	G	Q	G	1	C	1	T	1580	8097
4	966	FALSE	FALSE	FALSE	FALSE	D	K	G	Q	G	1	C	1	T	1580	649
5	967	FALSE	FALSE	FALSE	FALSE	D	K	G	Q	G	1	C	1	T	6962	1063
6	968	FALSE	FALSE	FALSE	FALSE	D	K	G	Q	G	1	C	1	T	8149	1319
7	969	FALSE	FALSE	FALSE	FALSE	D	K	G	Q	G	1	C	1	T	0	-
8	970	FALSE	FALSE	FALSE	FALSE	D	K	G	Q	G	1	C	1	T	1893	-
9	971	FALSE	FALSE	FALSE	FALSE	D	K	G	Q	G	1	C	1	T	1600	2194
10	972	FALSE	FALSE	FALSE	FALSE	D	K	G	Q	G	1	C	1	T	7367	1028
11	973	FALSE	FALSE	FALSE	FALSE	D	K	G	Q	G	1	C	1	T	8152	-
12	974	FALSE	FALSE	FALSE	FALSE	D	K	G	K	G	1	C	1	T	1901	1407
13	975	FALSE	FALSE	FALSE	FALSE	D	K	G	Q	G	1	C	1	T	1901	735
14	976	FALSE	FALSE	FALSE	FALSE	D	K	G	Q	G	1	C	1	T	1901	323

## Discussion

Antimicrobial drug resistance is a global emergency. The rapid development of antibiotic resistance in *N*. *gonorrhoeae* has the potential to reduce the clinical utility of nearly any antibiotic used for treatment within a few years of its introduction. Since 2007, CDC has discontinued recommending ciprofloxacin as anti-gonorrhea treatment 8 years since its initial recommendation [4]. With the advancement of newer, faster rapid molecular tests, there is a potential for clinicians to choose antibiotics discontinued for gonorrhea treatment based on the presence or absence of known genetic markers. This kind of targeted treatment potentially has the advantages of allowing physicians to select common and still effective antibiotics based on testing results [27–29].

Ciprofloxacin, a fluoroquinolone, is widely used to treat many bacterial infections such as pneumonia, meningitis, diarrhea, and urinary tract infections [30, 31]. This is the only class of antibiotic that directly inhibits bacterial DNA synthesis for gonorrhea treatment. It was the main choice for treating gonorrhea from 1998 to 2006 until resistance exceeded 5% and was removed from recommendations [2–4]. The main mutations conferring fluoroquinolone resistance are on the subunits of the DNA gyrase A, GyrA, and topoisomerase IV, ParC [33]. At the molecular level mutations conferring resistance are mainly at nucleotides C272 (amino acid S91) and A284 (amino acid D95) positions of *gyrA* and at the A259 (amino acid S87) location of the *parC* [28, 32–34].

Although ciprofloxacin has not been recommended for treating gonorrhea for over 10 years, the reported rates of ciprofloxacin resistant gonococcal isolates remain elevated. In the past ten years (2009–2017), the proportion of resistant isolates among sexually transmitted diseases (STD) clinics has increased steadily but remains below 40% [3, 9, 12, 25]. This also means that about 60% of uncomplicated gonococcal infections in the US are possibly still sensitive to ciprofloxacin. Therefore, ciprofloxacin may be considered for use in patients with confirmed, susceptible gonorrhea when recommended first-line therapy is not tolerated [33, 35]. This notion is especially facilitated by the fact that the ciprofloxacin resistance mechanisms utilized by *N*. *gonorrhoeae* are well defined and there are clear targets to be selected for developing rapid molecular tests [28, 29]. To this end, to conserve the usage of current first line antibiotics and in considering re-use of previously favored first line antibiotics, ciprofloxacin is an ideal candidate.

Here we created a panel of 14 gonococcal isolates which has the utility to serve multiple purposes for the scientific community. The mutations described in this panel provide a tool for scientists to develop molecular tests or validate existing tests. For instance, this panel could serve as an internal quality control for interlaboratory studies or external quality assurance for international collaborations. Finally, the isolates in this panel represent the susceptible, intermediate, and resistant phenotypes with respect to established gonococcal ciprofloxacin AST breakpoints.

In conclusion, this ciprofloxacin isolate panel has been extensively characterized based on its ciprofloxacin AST profile and the *gyrA* and *parC* genes sequences. This panel should be useful for developing ciprofloxacin susceptibility related tests or for quality assurance purposes [27, 28]. Availability of such tests for detecting ciprofloxacin susceptibility can provide clinicians with tools to improve antibiotic stewardship, potentially reducing costs, and save first line drugs for individuals that truly need it.
